# EHMT2‐mediated R‐loop formation promotes the malignant progression of prostate cancer via activating Aurora B

**DOI:** 10.1002/ctm2.70164

**Published:** 2025-01-06

**Authors:** Yuyang Zhang, Mingqin Su, Yiming Chen, Li Cui, Wei Xia, Renfang Xu, Dong Xue, Xiansheng Zhang, Xingliang Feng

**Affiliations:** ^1^ Department of Urology The First Affiliated Hospital of Anhui Medical University Hefei Anhui China; ^2^ Institute of Urology The First Affiliated Hospital of Anhui Medical University Hefei Anhui China; ^3^ Anhui Province Key Laboratory of Urological and Andrological Diseases Research and Medical Transformation Anhui Medical University Hefei Anhui China; ^4^ Department of Pathology The Second People's Hospital of Hefei, Hefei Hospital Affiliated to Anhui Medical University Hefei Anhui China; ^5^ Department of Urology The Third Affiliated Hospital of Soochow University Changzhou Jiangsu China; ^6^ Department of Urology The First People's Hospital of Changzhou Changzhou Jiangsu China

**Keywords:** Aurora B, CIN, CUL3, EHMT2

## Abstract

**Background:**

Chromosomal instability (CIN), a hallmark of cancer, is commonly linked to poor prognosis in high‐grade prostate cancer (PCa). Paradoxically, excessively high levels of CIN may impair cancer cell viability. Consequently, understanding how tumours adapt to CIN is critical for identifying novel therapeutic targets.

**Methods:**

Bioinformatic analyses were conducted to identify genes overexpressed in PCa tissues using The Cancer Genome Atlas (TCGA) and GEO datasets. Western blotting and immunohistochemistry assays were applied to determine the expression levels of euchromatic histone lysine methyltransferase 2 (EHMT2), pT232‐Aurora B and Cullin 3 (CUL3). The proliferation of cells was measured through CCK‐8 tests, clonogenesis and subcutaneous xenografts of human PCa cells in BALB/c nude mice. Live cell imaging, immunofluorescence (IF) and flow cytometry were used to confirm the role of EHMT2 in PCa cell mitosis. Co‐immunoprecipitation, Western blotting and IF assays further elucidated the underlying molecular mechanisms.

**Results:**

EHMT2 was highly expressed in metastatic PCa tissues exhibiting elevated CIN and was strongly associated with adverse clinical outcomes in patients with PCa. Silencing EHMT2 impaired cell division, inducing G2/M‐phase arrest and mitotic catastrophe in PCa cells. Mechanistically, EHMT2 is indispensable to ensure the full activation of Aurora B through centromeric R‐loop‐driven ATR–CHK1 pathway, with EHMT2 protein expression peaking during the G2/M‐phase. Moreover, CUL3 was identified as a binding partner of EHMT2, mediating its polyubiquitination and destabilising its protein levels.

**Conclusions:**

This study reveals a CUL3–EHMT2–Aurora B regulatory axis that safeguards accurate chromosome segregation in PCa cells, supporting the potential therapeutic application of EHMT2 inhibitors.

**Key points:**

- Euchromatic histone lysine methyltransferase 2 (EHMT2) is overexpressed in advanced prostate cancer, restraining catastrophic chromosomal instability (CIN) and enhancing cell fitness.
- EHMT2 functions via the centromeric R‐loop‐driven ATR–CHK1–Aurora B pathway to promote chromosomal stability.
- EHMT2 confers enzalutamide resistance via activating Aurora B.
- Cullin 3 (CUL3) promotes EHMT2 destabilisation via deubiquitination.

## Graphical abstract

1. Euchromatic histone lysine methyltransferase 2 (EHMT2) is overexpressed in advanced prostate cancer, restraining catastrophic chromosomal instability (CIN) and enhancing cell fitness.
2. EHMT2 functions via the centromeric R‐loop‐driven ATR–CHK1–Aurora B pathway to promote chromosomal stability.
3. EHMT2 confers enzalutamide resistance via activating Aurora B.
4. Cullin 3 (CUL3) promotes EHMT2 destabilisation via deubiquitination.

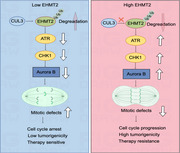

## 1 INTRODUCTION

Prostate cancer (PCa) is a common tumour among men globally, with androgen receptor (AR) signalling playing a pivotal role in its pathogenesis.^1^, ^2^ Androgen deprivation therapy, aimed at inhibiting AR signalling, remains the primary treatment for patients with PCa.^2^ Despite initial efficacy, this approach eventually leads to the progression to metastatic castration‐resistant prostate cancer (mCRPC), an aggressive and currently incurable subtype.^3^, ^4^ Consequently, innovative therapy approaches are therefore desperately needed, especially for mCRPC.

Chromosomal instability (CIN), a key type of genomic instability characterised by continuous chromosomal deletion or gain and the resultant aneuploidy, is linked to increased tumour variation, metastases and resistance to therapies across multiple cancer types.^3^, ^5^, ^6^ Genomic dataset analyses reveal that metastatic PCa shows significantly higher levels of chromosomal alterations and instability compared to primary tumours.^7^, ^8^ Additionally, meta‐analyses of patients with PCa indicate that those with elevated CIN and/or intratumour heterogeneity experience reduced survival compared to patients with chromosomally stable cancers. Notably, a high CIN state correlates with advanced tumour stages and chemotherapy resistance in PCa. Interestingly, while CIN can drive tumour evolution, excessive CIN levels can inflict catastrophic DNA damage, leading to intolerable genomic instability that compromises cancer cell viability.^9^ This suggests that tumour cells must employ adaptive strategies to manage CIN and maintain cellular equilibrium.^5^, ^10^, ^11^ In PCa, clinical investigations correlate aneuploidy and CIN with aggressive, lethal disease; however, the underlying drives, adaptive mechanisms and therapeutic implications of CIN in PCa remain insufficiently explored.

Aurora B kinase, a serine–threonine kinase, acts a crucial role in mitotic spindle formation and accurate chromosome segregation.^12^, ^13^ Dysregulation of this kinase and related pathways has been implicated in various malignancies, including liver, leukaemia and breast cancers.^14^, ^15^, ^16^, ^17^ For example, c‐Myc promotes Aurora B expression, and the resulting increase in Aurora kinase activity drives the progression of B‐cell lymphoma.^14^ Additionally, recent studies demonstrate that the ATR–CHK1 pathway activates Aurora B, functioning as a non‐genetic tolerance pathway that ensure chromosome segregation fidelity and conferring resistance to anti‐EGFR treatment in lung cancer.^18^, ^19^, ^20^ Aurora B has also been implicated in resistance against specific chemotherapy drugs, including aromatase inhibitors in breast carcinoma,^21^ paclitaxel in lung cancer^22^ and vemurafenib in melanoma.^23^ Although Aurora B expression is elevated in advanced prostate tumours,^5^ its precise role in PCa remains unclear.

Euchromatic histone lysine methyltransferase 2 (EHMT2) is the main enzyme responsible for the monomethylation and dimethylation of histone H3K9.^24^, ^25^ EHMT2 knockout in mice results in embryonic lethality because of severe growth defects.^26^ Elevated EHMT2 expression is commonly reported in multiple human malignancies and is strongly related to tumour progression, metastasis and poor prognosis.^27^, ^28^, ^29^ EHMT2 depletion in cancer cells impedes cell growth and significantly reduces tumour expansion by modulating several cellular processes, such as transcription, DNA damage response and repair and RNA processing.^24^, ^30^, ^31^ Despite growing studies on EHMT2 in malignancy, substantial gaps remain, particularly in knowing its role in CIN regulation and the mechanisms regulating its protein levels.

This study identified the Cullin 3 (CUL3)–EHMT2–Aurora B signalling cascade as essential for PCa cells to manage CIN during mitosis. Clinical dataset analyses and tissue samples revealed that EHMT2 expression was considerably upregulated in advanced PCa samples and along with poor outcome. EHMT2 deficiency disrupted chromosome alignment, bi‐orientation and segregation, sensitising PCa cells to enzalutamide (ENZ) via the centromeric R‐loop‐driven ATR–CHK1 pathway. Additionally, CUL3 interacted with EHMT2, mediating its polyubiquitination and destabilising EHMT2 protein levels. Collectively, our findings underscore the prognostic value of elevated EHMT2 expression in PCa and shed light on the novel mechanisms by which EHMT2 drives PCa progression and drug resistance.

## 2 RESULTS

### 2.1 EHMT2 is overexpressed in advanced PCa tissues

**FIGURE 1:**
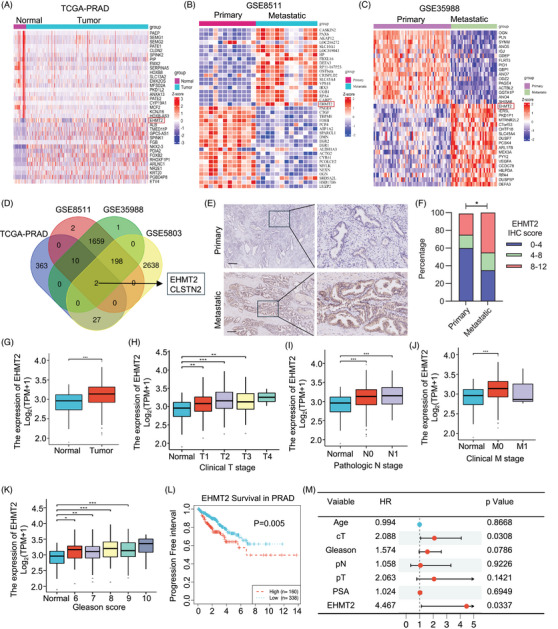
Euchromatic histone lysine methyltransferase 2 (EHMT2) is highly expressed in prostate cancer (PCa) tumours and correlated with poor clinical outcomes of patients with PCa. (A–C) Heatmaps display the top 20 differentially gene expression in indicated publicly available datasets (The Cancer Genome Atlas [TCGA]‐PRAD, GSE8511, GSE35988, GSE5803). The red square highlights EHMT2. (D) Venn diagram showing the common upregulated genes across the four datasets. (E, F) Representative EHMT2 immunohistochemistry images and quantification of EHMT2 protein levels in metastatic (*n* = 20) and non‐metastatic (*n* = 18) prostate cancer specimens. Scale bar, 100 µm. (G) EHMT2 expression levels in the GSE114740. (H–K) Correlation of EHMT2 mRNA expression with tumour stage (H), lymph node metastasis (I), distant metastasis (J) and Gleason score (K) in patients with PCa from the TCGA database. N0, no lymph node metastasis; N1, nearby lymph node metastasis; N2, distant lymph node metastasis; M0, no distant metastasis; M1, distant metastasis. (L) Kaplan–Meier survival curves for Disease‐free‐survival (DFS) based on EHMT2 expression in the TCGA‐PRAD cohort. (M) Multivariate analysis of the outcomes of patients with PRAD based on a COX regression model, incorporating factors associated with clinical outcomes. *p < .05, **p < .01, ***p < .001.

To elucidate potential mechanisms contributing to PCa progression, this study analysed expression profile changes in The Cancer Genome Atlas (TCGA)‐PRAD and three GEO datasets that include cancerous and adjacent normal tissues as well as primary and metastatic PCa samples (TCGA‐PRAD, GSE8511, GSE35988 and GSE5803). Differential expression analysis identified the top 20 most significantly differentially expressed genes (DEGs), which were visualised in heatmaps (Figures 1A–C and S1A). Notably, EHMT2 and CLSTN2 consistently showed elevated expression across all datasets (Figure 1D). This observation was further validated in a cohort of 20 primary and 18 metastatic PCa samples, where EHMT2 protein levels were significantly higher in metastatic tissues, whereas CLSTN2 expression remained largely unchanged (Figures 1E,F and S1B). Other available datasets also supported the association between EHMT2 expression and advanced PCa (Figure 1G). Given recent studies linking EHMT2 to PCa tumourigenesis,^32^ we sought to further explore in detail the function of EHMT2 upregulation in advanced PCa. Consistently, both mRNA and protein levels of EHMT2 were markedly higher in advanced PCa cell lines (Figure S1C–E). TCGA data analysis demonstrated a robust association between EHMT2 expression and pathological features in PCa, with elevated EHMT2 levels correlating associating strongly with tumour size, lymph node and distant metastases, stage and Gleason score (Figure 1H–K). Clinically, increased EHMT2 mRNA expression appears to be associated with poorer survival in patients with PCa (Figure 1L). Furthermore, multivariate Cox regression analysis indicated that EHMT2 expression may function as an independent indicator of prognosis in PCa (Figure 1M). In summary, our study identifies EHMT2 as highly expressed in advanced PCa, where it serves as a poor prognostic indicator.

### 2.2 EHMT2 is required for the progression of mitosis

**FIGURE 2:**
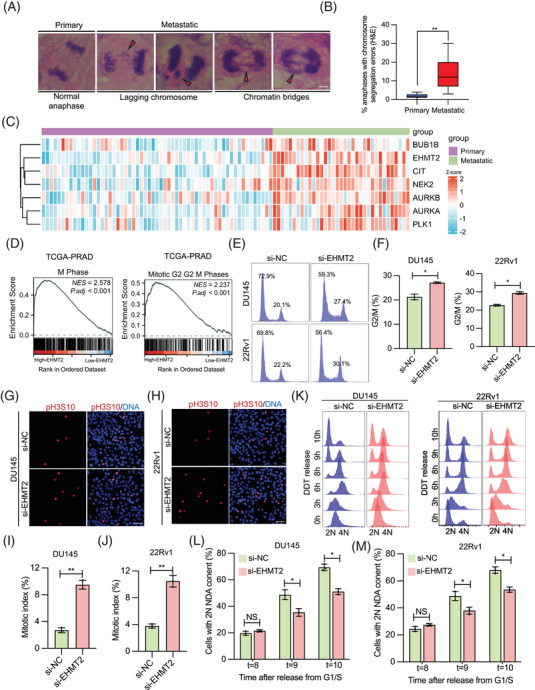
Euchromatic histone lysine methyltransferase 2 (EHMT2) deletion leads to significant mitotic cell accumulation. (A, B) Representative images and quantification of anaphases showing chromosome segregation errors in the tissue samples of patients with primary and metastatic prostate cancer (PCa). Arrows indicate lagging chromosomes or chromatin bridges. Scale bar, 5 mm. (C) Heatmaps showing transcriptome data for mitotic kinases and EHMT2 in publicly available PCa datasets. (D) Gene set enrichment analysis (GSEA) revealing the correlation between EHMT2 and G2/M in the TCGA_PRAD dataset. (E, F) Flow cytometry analysis of cell cycle effects after transfected short interfering RNA (siRNA) against EHMT2 for 48 h. (G–J) Immunofluorescence visualisation of mitotic cells. DU145 and 22Rv1 cells were transfected siRNA against EHMT2 for 48 h, subsequently, stained for pH3S10 (red) and DAPI (blue). Scale bars, 50 µm. (K–M) Analysis of DNA content by flow cytometry in DU145/si‐NC, DU145/si‐EHMT2, 22Rv1/si‐NC and 22Rv1/si‐EHMT2 cells synchronised at the G1/S boundary via double‐thymidine block, then released. *p < .05, **p < .01.

Metastatic PCa is consistently associated with high levels of CIN.^3^, ^33^ In alignment with these findings,^5^ our analysis revealed that metastatic PCa tissues exhibit elevated CIN compared to primary tumours, as indicated by an increased frequency of anaphase cells displaying chromosomal mis‐segregation (Figure 2A,B). Given that CIN predominantly is driven by mistakes in mitotic chromosome segregation, this study investigated whether EHMT2 plays a role in pathways that preserve chromosomal stability during mitosis in advanced PCa. Our findings indicate that EHMT2, along with several mitotic kinases, is markedly upregulated in metastatic PCa tissues relative to primary tumours (Figure 2C). Gene set enrichment analysis (GSEA) in the TCGA dataset further revealed a positive connection involving EHMT2 expression and cell cycle phases M and G2/M (Figure 2D). This result was further validated by RNA‐Seq analysis, comparing gene expression in cells overexpressing EHMT2 with those containing an empty vector control (Figure S2A,B). To explore EHMT2's function in mitosis, the mitotic index was assessed in DU145 and 22Rv1 cells. Fluorcence‐activated cell sorting (FACS) analysis showed a higher percentage of cells in the G2/M‐phase upon EHMT2 deletion (Figure 2E,F). Correspondingly, immunofluorescence (IF) staining revealed elevated levels of pH3S10‐postive cells in EHMT2‐deficient cells (Figure 2G–J), supporting EHMT2's role in promoting mitotic progression. This mitotic delay was additionally observed in synchronised cells as they proceeded through mitosis after being released from a double‐thymidine block, evidenced by a delay in forming daughter cells with a 2N DNA content in FACS analysis (Figure 2K–M). Together, these findings imply that EHMT2 is crucial for the efficient progression of mitosis.

### 2.3 EHMT2 deficiency leads to deleterious CIN and impairs proliferation

**FIGURE 3:**
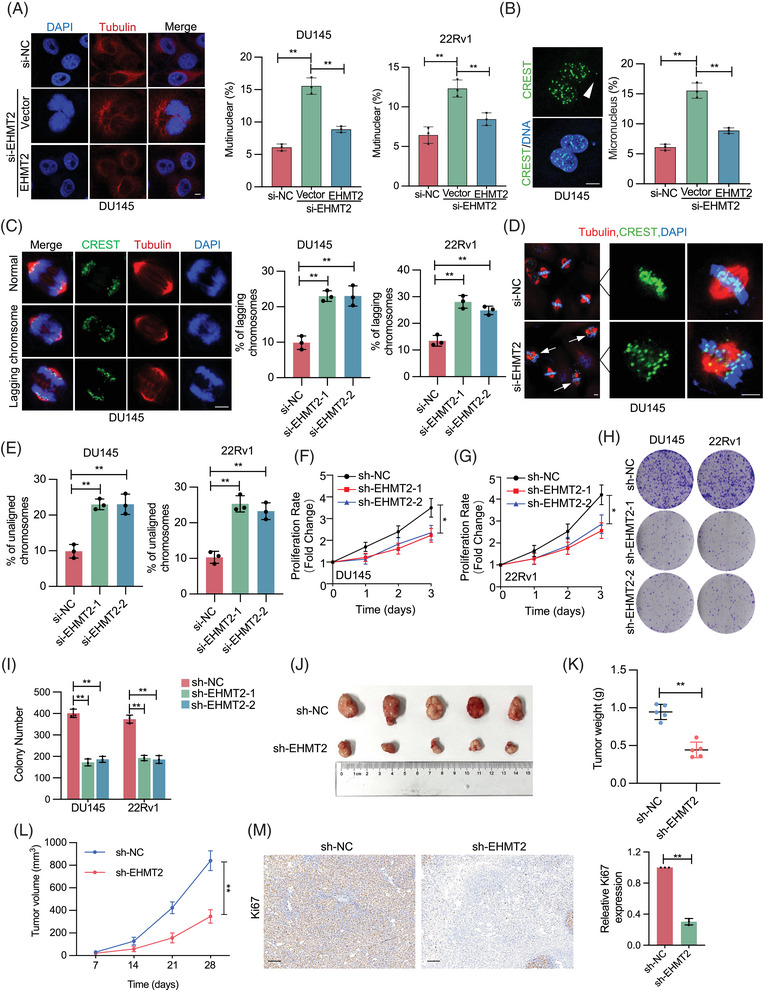
Euchromatic histone lysine methyltransferase 2 (EHMT2) deficiency leads to aberrant mitosis. (A) Immunofluorescence staining with anti‐tubulin was conducted on DU145 and 22Rv1 cells transfected with either EHMT2 short interfering RNA (siRNA) or control siRNA for 36 h. Nuclei were stained with DAPI. Quantitative analysis of multinuclear cells among control, EHMT2 knockdown and EHMT2 rescue cells is presented. Scale bar, 5 mm. (B) Percentage of micronuclei in DU145 cells transfected with control or EHMT2 siRNA for 48 h. Micronuclei were stained for centromeres (CREST) and DNA. Quantitative comparison of micronuclei in control, EHMT2 knockdown and EHMT2 rescue cells is shown. Scale bar, 5 mm. (C) Percentage of anaphase DU145 and 22Rv1 cells (>100 anaphases analysed per condition) with lagging chromosomes following transfection with either EHMT2 siRNA or control siRNA for 48 h. Scale bar, 5 mm. (D, E) Percentage of DU145 and 22Rv1 cells (>100 metaphases analysed per condition) with chromosome misalignment following transfection with either EHMT2 siRNA or control siRNA for 48 h. Scale bar, 5 mm. (F, G) CCK8 assay to assess proliferation of DU145 and 22Rv1 cells with EHMT2 knockdown. (H, I) Colony formation assay performed on DU145 and 22Rv1 cells transfected with either EHMT2 siRNA or control siRNA for 48 h. (J–L) Xenografts derived from DU145‐shEHMT2 and respective controls (*n*  =  5). Tumour volume was recorded at indicated times to establish tumour weight (K) and growth curves (L). (M) Ki67 immunohistochemistry (IHC) staining in xenograft tissues. NS, no significant; *p < .05; **p < .01; ***p < .001.

To investigate EHMT2's role in maintaining mitotic integrity, EHMT2 was knocked down via short interfering RNA (siRNA) in DU145 and 22Rv1 cells, with mitotic division subsequently monitored. EHMT2 knockdown markedly increased the incidence of multinuclear cells, while the reintroduction of EHMT2 expression in EHMT2‐siRNA cells restoration multinuclear cell levels to near‐normal (Figures 3A and S3A). Depletion of EHMT2 also resulted in a significant rise in micronuclei levels (Figure 3B), demonstrating that EHMT2 is a crucial modulator of genomic stability during mitosis. To further assess mitotic abnormalities caused by EHMT2 loss, chromosome segregation was analysed in EHMT2‐depleted DU145 and 22Rv1 cells. As demonstrated in Figures 3C–E and S3B, EHMT2 depletion led to increased occurrences of lagging chromosomes and chromosomal misalignment, indicating that EHMT2 loss induces mitotic catastrophe.

For a direct examination of EHMT2's role in PCa cells, EHMT2 was knocked down in DU145 and 22Rv1 cells using siRNA. Proliferation assays revealed that EHMT2‐depleted cells exhibited slower proliferation compared to those transfected with scramble siRNA (Figure 3F,G). Consistently, colony formation assays indicated that stable EHMT2 knockdown impaired colony formation ability (Figure 3H,I). To evaluate the in vivo impact of EHMT2 on PCa proliferation, DU145 or 22Rv1 cells stably transfected with EHMT2‐shRNA or scramble‐shRNA were injected subcutaneously into BALB/c nude mice. Tumours in the EHMT2 knockdown group showed significant reductions in size and weight relative to the control group. (Figures 3J–L and S3C–F). Additionally, immunohistochemistry (IHC) staining revealed a marked decrease in Ki67 expression following EHMT2 knockdown (Figure 3M). Notably, EHMT2‐shRNA DU145 xenograft tumours showed higher anaphases with chromosome segregation mistakes relative to control tumours (Figure S3G). Collectively, EHMT2 deficiency leads to deleterious CIN in PCa cells and impairs cellular proliferation.

### 2.4 EHMT2 is essential for the Aurora B kinase

**FIGURE 4:**
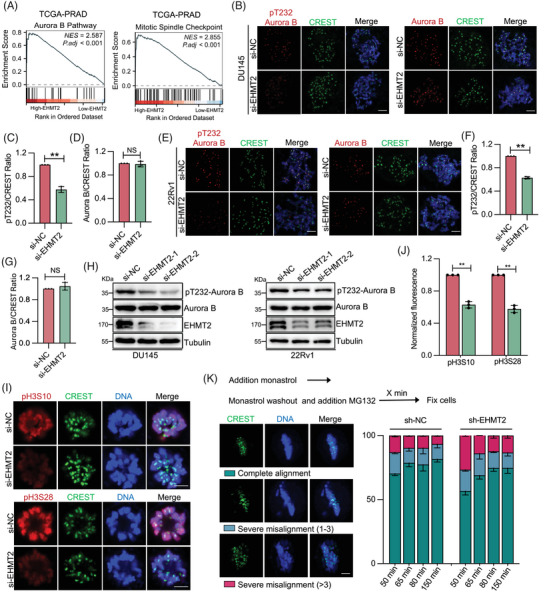
Euchromatic histone lysine methyltransferase 2 (EHMT2) is essential for Aurora B kinase activity. (A) Gene set enrichment analysis (GSEA) revealing the correlation between EHMT2 and Aurora B/Mitotic Spindle Checkpoint pathways in the TCGA_PRAD dataset. (B–D) DU145 cells transfected with control or EHMT2 short interfering RNA (siRNA) were treated with nocodazole for 4 h. Mitotic cells were fixed with 4% paraformaldehyde and stained with pT232‐Aurora B (C), Aurora B (D) and CREST antibodies. Scale bar, 5 mm. (E–G) 22Rv1 cells transfected with control or EHMT2 siRNA were treated with nocodazole for 4 h. Mitotic cells were fixed with 4% paraformaldehyde and stained with pT232‐Aurora B (F), Aurora B (G) and CREST antibodies. Scale bar, 5 mm. (H) Immunoblotting of mitotic DU145 and 22Rv1 cell lysates obtained from cells transfected with control or EHMT2 siRNA, using the indicated antibodies. (I, J) Representative images of p‐H3 S10 and p‐H3 S28 in mitotic DU145 cells transfected with control or EHMT2 siRNA for 48 h. Cells were treated with STLC for 4 h. Scale bar, 5 mm. (K) Quantification of chromosome alignment categories (%) following monastrol washout into MG132 at various time points, with a schematic depiction of the experimental setup at the top. Scale bar, 5 mm. NS, no significant; *p < .05; **p < .01; ***p < .001.

Following the establishment of EHMT2's role in mitosis, downstream effectors and mechanisms were examined to elucidate its mitotic function. GSEA of the TCGA‐PRAD database indicated a strong connection between high EHMT2 expression and both the mitotic spindle assembly and Aurora B pathways (Figure 4A). As anticipated, EHMT2 depletion in nocodazole‐arrested prometaphase DU145 and 22Rv1 cells significantly reduced the centromeric distribution of Thr232‐phosphorylated Aurora B (Aurora B‐pT232), which is essential for Aurora B's full activation^34^, ^35^ (Figure 4B–G). In contrast, EHMT2 depletion did not alter the distribution of Aurora B itself or additional components of the chromosomal passenger complex (CPC), including inner centromere protein (INCENP) and Survivin (Figure S4A–C), indicating that EHMT2 specifically impairs Aurora B activation at centromeres without affecting its localisation. Supporting this, EHMT2‐depleted mitotic cells demonstrated a decrease in global Aurora B‐pT232 levels, while total Aurora B protein levels maintained steady (Figure 4H). Consistent with this, EHMT2‐depleted mitotic cells have reduced Aurora B‐dependent phosphorylation of histone H3 at Ser10 (pH3S10) and Ser28 (pH3S28).

Aurora B kinase is essential for correcting erroneous kinetochore–microtubule (KT–MT) attachments. To determine whether EHMT2 depletion affects this correction process, a monastrol washout assay was conducted (Figure 4K). Cells were administered using the Eg5 inhibitors to arrest them in mitosis with monopolar spindles, thereby increasing the incidence of erroneous KT–MT attachments.^36^ Cells were then allowed to progress through mitosis at various time points (50–150 min) following release from the monastrol treatment, with the proteasome inhibitor MG132 added to avoid anaphase beginning. The proportion of cells displaying correctly aligned against misaligned chromosomes was quantified to be an indicator of error correction efficiency. After 50 min, approximately 70% of wild‐type (WT) cells had achieved full alignment, in contrast to approximately 49% of EHMT2 KD cells. By 150 min, WT cells reached maximal alignment (±85%), while EHMT2‐depleted cells recovered to around 76%, suggesting that error correction occurs but at a slower pace. These results demonstrate that defects in Aurora B kinase activity at centromeres as the likely cause of lagging chromosomes observed with EHMT2 depletion.

### 2.5 The EHMT2–ATR–CHK1 axis controls Aurora B activity

**FIGURE 5:**
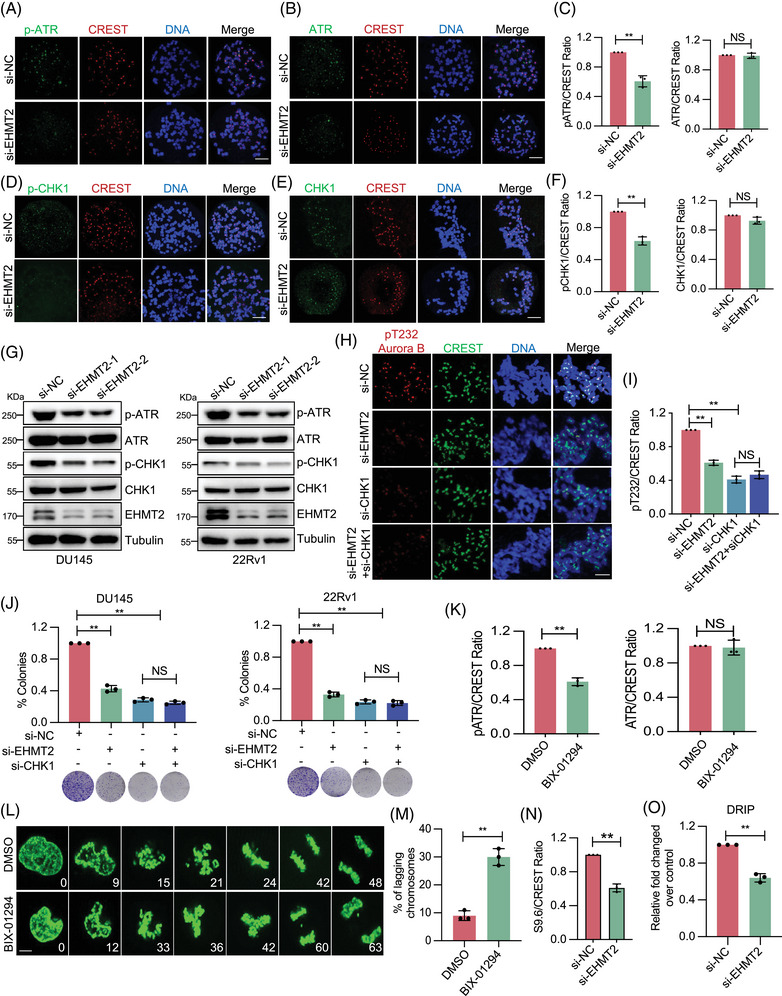
Euchromatic histone lysine methyltransferase 2 (EHMT2) promotes Aurora B activation via the centromeric R‐loop‐driven ATR–CHK1 pathway. (A–C) Fluorescence intensities of centromeric p‐ATR and ATR in mitotic DU145 cells. Indicated cells were treated with nocodazole for 6 h to facilitate chromosome spreading. Scale bar, 5 mm. (D–F) Fluorescence intensities of centromeric p‐CHK1 and CHK1 in mitotic DU145 cells. Indicated cells were treated with nocodazole for 6 h to promote chromosome spreading. Scale bar, 5 mm. (G) Immunoblotting of mitotic DU145 and 22Rv1 cell lysates obtained from cells transfected with control or EHMT2 short interfering RNA (siRNA), using the indicated antibodies. (H‐J) Quantification of pT232‐Aurora B levels and colony formation in cells transfected with the indicated siRNAs. Scale bar, 5 mm. (K) Fluorescence intensity of centromeric p‐ATR, ATR and CREST in prometaphase DU145 cells treated with DMSO or the EHMT2 inhibitor BIX‐01294. (L‐M) Representative images of GFP‐H2B‐HeLa cells treated with DMSO or the EHMT2 inhibitor BIX‐01294 immediately after mitotic entry. Scale bar, 5 mm. (M) Fluorescence intensity of centromeric S9.6 in prometaphase DU145 cells. (O) Quantitative polymerase chain reaction (PCR) analysis of DNA–RNA hybrid immunoprecipitation (DRIP) in mitotic DU145 cells. NS, no significance; *p < .05; **p < .01; ***p < .001.

We next explored how EHMT2 regulates Aurora B activation in mitosis. It is known that EHMT2 can facilitate RPA–ssDNA complex formation in response to DNA damage, thus activating the ATR–CHK1 pathway.^31^ Interesting, recent studies have highlighted a role for ATR–CHK1 in centromeric function and mitotic spindle assembly,^18^ prompting an inquiry into whether the ATR–CHK1 pathway might mediate EHMT2's function in Aurora B activation. To test this, the impact of EHMT2 on ATR centromeric localisation and kinase activity was examined. As depicted in Figures 5A–C and S5A,B, ATR localisation at centromeres remained unchanged in EHMT2‐depleted DU145 and 22Rv1 cells arrested in prometaphase with nocodazole treatment, although ATR autophosphorylation (p‐ATR T1989) was significantly reduced. Additionally, while centromeric CHK1 localisation was unaffected in EHMT2‐depleted cells, ATR‐phosphorylated CHK1 (CHK1 S317) levels decreased (Figures 5D–F and S5C,D). These results, supported by Western blotting analysis (Figure 5G), suggest that the ATR–CHK1 pathway plays a role in EHMT2‐mediated Aurora B activation.

To rigorously determine whether EHMT2's facilitation of Aurora B activation is mediated via ATR–CHK1 signalling, CHK1 was inhibited independently and in conjunction with EHMT2 inhibition. Results indicated that CHK1 inhibition caused a more pronounced decrease in p‐Aurora B levels than EHMT2 depletion alone, while combined inhibition of EHMT2 and CHK1 did not further reduce p‐Aurora B (Figures 5H,I and S6A). Furthermore, CHK1 knockdown alone and in combination with EHMT2 knockdown resulted in comparably severe mitotic aberrations and proliferation defects (Figures 5J and S6B), supporting that EHMT2 promotes Aurora B activation via CHK1.

To eliminate the possibility that this phenotype resulted from EHMT2 disruption within the DNA repair pathway, a small‐molecule inhibitor of EHMT2 (BIX‐01294) was introduced into cultured cells immediately after mitotic entry. Treatment with EHMT2i during mitosis reduced p‐Aurora B levels and enhanced the frequency of lagging chromosomes (Figure 5L,M), suggesting that EHMT2 directly participates in mitosis to ensure accurate chromosome segregation. As expected, BIX‐01294 treatment impaired cell proliferation and elevated chromosome mis‐segregation in DU145 and 22RV1 cells (Figure S7A–C).

Subsequently, the activation of the ATR–CHK1 signalling pathway was examined by EHMT2 at centromeres during mitosis. Given that ATR activation at centromeres is driven by R‐loops, this study investigated whether EHMT2's role in promoting ATR–CHK1 signalling involved R‐loops.^18^ EHMT2‐depleted cells exhibited reduced centromeric S9.6 staining, which specifically detects DNA–RNA hybrids (Figure 5N). To verify this finding, DNA–RNA hybrid immunoprecipitation (DRIP) was performed using an RNaseH1 mutant (RNaseH1 D210N) that binds but does not cleave DNA–RNA hybrids due to its RNase‐dead mutation. Consistently, a significant reduction in DNA–RNA hybrid signals was observed at centromeres in EHMT2‐depleted cells (Figures 5O and S8A), suggesting that EHMT2 supports Aurora B activation and chromosome segregation through R‐loop stabilisation. Furthermore, co‐localisation of p‐ATR, p‐CHK1 and p‐Aurora B was impaired upon RNaseH1 WT expression (Figure S8B–D), which notably increased the frequency of lagging chromosomes (Figure S8E). These results underscore the critical role of EHMT2‐mediated R‐loops in ATR–CHK1–Aurora B pathway activation.

### 2.6 EHMT2 is upregulated during mitosis

**FIGURE 6:**
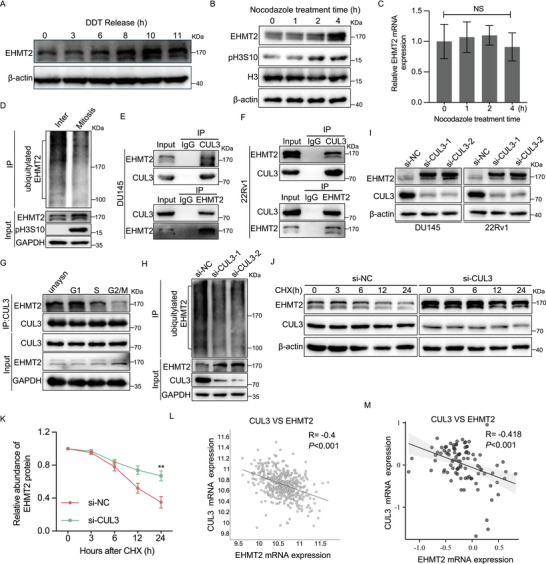
Cullin 3 (CUL3) promotes euchromatic histone lysine methyltransferase 2 (EHMT2) protein degradation via ubiquitination. (A) DU145 cells were synchronised at the G1/S boundary using double‐thymidine blocking, then released, and EHMT2 protein levels were analysed by Western blotting. (B) Western blotting analysis of EHMT2 protein levels in DU145 cells treated with .66 µM nocodazole (NOC) for the indicated time points. (C) EHMT2 mRNA levels analysed by real time‐polymerase chain reaction (RT‐PCR) in DU145 cells following treatment with .66 µM nocodazole (NOC) for the specified times. (D) Ubiquitination of EHMT2 in interphase and mitotic cells assessed by Western blotting analysis. (E, F) Immunoprecipitation performed on DU145 and 22Rv1 cell lysates with indicated antibodies, followed by immunoblotting with the specified antibodies. (G) Immunoprecipitation and Western blotting analysis showing the interaction between EHMT2 and CUL3 under the specified conditions. (H) Ubiquitination of EHMT2 in DU145 cells with CUL3 knockdown, analysed by immunoprecipitation and Western blotting. Cells were treated with MG132 (20 µM for 4 h) before harvesting. (I) DU145 and 22Rv1 cells transfected with indicated short interfering RNAs (siRNAs), with Western blotting analysis performed to assess EHMT2 and CUL3 protein levels. (J, K) EHMT2 protein levels in DU145 cells following CUL3 downregulation at specified time points after cycloheximide (CHX, 20 µM) treatment. CHX is a protein synthesis inhibitor that is commonly used to observe the degradation of existing proteins and determine the half‐life of specific proteins. (L, M) Correlation analysis of EHMT2 and CUL3 expression based on The Cancer Genome Atlas (TCGA)‐PRAD and GSE35988 datasets. NS, no significance; *p < .05; **p < .01; ***p < .001.

To elucidate the specific role of EHMT2 in mitotic progression, EHMT2 expression across the cell cycle was examined. DU145 cells were synchronised at the G1/S boundary using double‐thymidine blocking and subsequently released to progress through the cell cycle. Western blotting analysis revealed that EHMT2 levels remained stable during the G1/S‐phase, comparable to those in asynchronised cells. Notably, a substantial increase in EHMT2 was observed in M‐phase cells (approximately 10 h post‐release from double‐thymidine block) and in cells arrested in the M‐phase with nocodazole treatment (Figure 6A,B). To elucidate the molecular mechanisms driving EHMT2 upregulation in mitotic cells, mRNA levels of EHMT2 were measured. Real time‐polymerase chain reaction (RT‐PCR) analysis indicated that EHMT2 mRNA levels in nocodazole‐treated cells were similar to those in untreated cells (Figure 6C), implying that the increase in EHMT2 expression is not attributable to transcriptional upregulation.

### 2.7 CUL3 is an E3 ligase mediating polyubiquitination of EHMT2

Since ubiquitination is recognised as a key post‐translational modification regulating protein stability, EHMT2 ubiquitination was analysed upon mitotic entry. As shown in Figure 6D, EHMT2 ubiquitination occurred primarily in interphase, with markedly lower levels in the M‐phase. To identify the E3 ligases contributing to EHMT2 ubiquitination, potential EHMT2‐interacting proteins were examined based on a previous study.^37^ CUL3 emerged as a putative binding partner for EHMT2, and this interaction was confirmed through reciprocal immunoprecipitation of endogenous EHMT2 and CUL3 (Figure 6E,F), suggesting that CUL3 likely serves as an E3 ligase for EHMT2. Notably, this interaction was notably diminished in G2/M‐synchronised cells exposed to nocodazole (Figure 6G).

To further investigate CUL3's regulatory influence on EHMT2, CUL3 expression levels were modulated, and EHMT2 protein levels were assessed. Silencing CUL3 led to a marked increase in EHMT2 protein levels and a significant reduction in EHMT2 polyubiquitination (Figure 6H,I). Consistent with these findings, siRNA‐mediated CUL3 depletion significantly extended the half‐life of EHMT2 (Figure 6J,K). Furthermore, findings from the TCGA‐PRAD and GSE35988 databases demonstrated a strong negative correlation between CUL3 and EHMT2 expression levels (Figure 6L,M). These results collectively indicate that CUL3 functions as the E3 ligase responsible for EHMT2 polyubiquitination and degradation.

### 2.8 EHMT2 enhances prostate cancer cell resistance to enzalutamide through controls Aurora B activity

Given the biological role of EHMT2 in promoting cell cycle progression, enhancing cell viability and supporting mitosis in PCa cells, its potential involvement in facilitating drug resistance was investigated. ENZ, a first‐line therapy for CRPC,^38^, ^39^ faces significant resistance issues linked to poor prognosis, making ENZ resistance a challenging clinical problem. Therefore, the influence of EHMT2 on ENZ resistance was examined.

**FIGURE 7:**
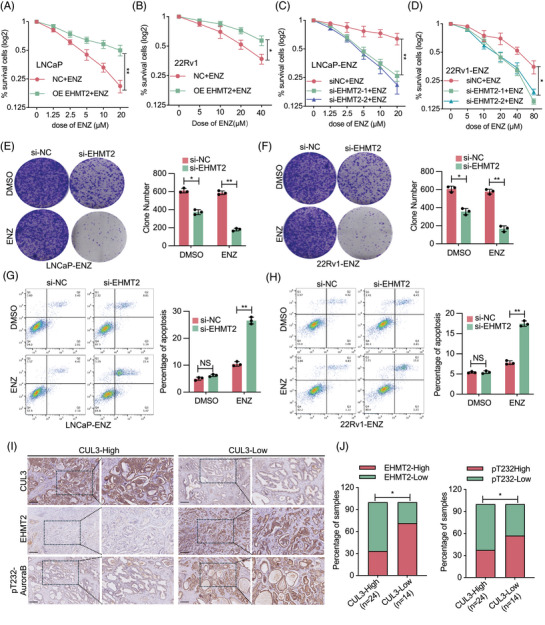
Euchromatic histone lysine methyltransferase 2 (EHMT2) enhances cell resistance to enzalutamide (ENZ) in vitro. (A, B) Control and EHMT2‐overexpressing LNCaP/22Rv1 cells were treated with increasing doses of ENZ, with cell survival assessed via CCK‐8 assay. (C, D) Control and EHMT2‐knockdown (using short interfering RNA [siRNA]) LNCaP‐ENZ/22Rv1‐ENZ cells were treated with increasing doses of ENZ, and cell survival rates were measured using CCK‐8 assay. (E‐F) Cell proliferation determined in indicated cells with ENZ treatment. LNCaP‐ENZ/22Rv1‐ENZ cells were transiently transfected as indicated. Following treatment with ENZ, the cells were subjected to colony formation assay. (G, H) Flow cytometry analysis of cell apoptosis for cells transfected with EHMT2 siRNAs or control siRNAs under the treatment of DMSO or ENZ. (H, I) Proposed model illustrating the mechanisms of the Cullin 3 (CUL3)–EHMT2–Aurora B axis in prostate cancer. NS, no significance; *p < .05; **p < .01; ***p < .001.

LNCaP and 22Rv1 cells were transfected with either a blank or EHMT2‐overexpressing plasmids and subsequently treated with increasing doses of ENZ. In LNCaP and 22Rv1 cells, cell numbers declined significantly in response to rising ENZ doses; however, EHMT2 overexpression partially mitigated ENZ‐induced cytotoxicity (Figure 7A,B). To further assess EHMT2's impact, EHMT2 was knocked down in ENZ‐resistant LNCaP and 22Rv1 cells. Cell proliferation of transfected cells exposed to ENZ or DMSO was assessed by clonogenesis assays. Our findings demonstrated that inhibiting EHMT2 in combination with ENZ treatment reduced the number of colonies generated by ENZ‐resistant cells (Figure 7C–F). Consistent with these findings, Annexin V‐FITC and propidium iodide (PI) staining revealed that the combined administration of siEHMT2 and ENZ led to considerable apoptosis or necrosis of ENZ‐resistant cells (Figure 7G,H). These findings imply that elevated EHMT2 expression makes PCa cells more resistant to ENZ.

To determine if EHMT2's role in promoting ENZ resistance involved Aurora B signalling, EHMT2 was knocked down alone or in combination with Aurora B knockdown using siRNA. As shown in Figure S9, knocking down both EHMT2 and Aurora B had no cumulative effect on ENZ sensitivity, suggesting that these proteins enhance ENZ resistance through a shared pathway.

### 2.9 CUL3–EHMT2–Aurora B axis triggers the progression of prostate cancer

Additionally, the relationship between CUL3, EHMT2, Aurora B‐pT232 and PCa progression was explored. Given that CUL3 regulates EHMT2 stability, protein expression levels of CUL3, EHMT2 and Aurora B‐pT232 were evaluated in 38 PCa tissue samples using IHC, with staining intensity as a scoring criterion. IHC analysis revealed that patients with high EHMT2 expression exhibited comparatively low CUL3 and high Aurora B‐pT232 expression, indicating a possible link between these markers and PCa development.

## 3 DISCUSSION

Metastatic PCa remains an incurable, lethal disease.^2^, ^40^ Previous studies have shown that PCa accumulates significant genomic alterations as the disease advances, with metastatic tumours displaying the greatest amounts of CIN among all metastatic cancer types.^3^, ^41^ Comparative analyses between castration‐sensitive prostate cancer (CSPC) and mCRPC indicate that CIN serves as a predictor of advanced metastatic stage, poor prognosis and therapeutic resistance.^10^ While CIN promotes malignant progression by increasing cellular heterogeneity, high CIN levels can paradoxically undermine tumour cell survival, indicating that tumour cells evolve adaptive mechanisms to balance instability with viability. This study demonstrates that EHMT2 expression is associated with elevated CIN levels and is essential for PCa cells to maintain chromosome mis‐segregation rates within a range that maximises viability. EHMT2 depletion induces multiple mitotic deficiencies, including chromosome misalignment and lagging chromosomes, resulting in severe genomic instability. Additionally, EHMT2 enables cells to tolerate CIN through a centromeric R‐loop‐driven ATR–CHK1 pathway that activates Aurora B kinase.

Aurora B, a serine/threonine kinase, is critical for mitosis and cytokinesis^12^, ^13^, ^42^, ^43^ and exhibits increased activation across various cancers, including advanced PCa that adapts to CIN.^44^, ^45^ Aurora B activation is initiated by its binding to the INCENP, stabilising Aurora B's active G‐loop conformation and opening its catalytic cleft. Aurora B subsequently phosphorylates the Thr‐Ser‐Ser motif within INCENP's IN box, a step essential for its full activation.^44^, ^46^, ^47^ Aurora B activation is also regulated by extrinsic factors, including mitotic kinases such as MPS1, HASPIN, PLK1 and the centromeric R‐loop‐driven ATR–CHK1 pathway.^47^, ^48^, ^49^, ^50^ However, the precise activation mechanism of Aurora B in PCa and the involvement of other molecules remain unclear. Here, EHMT2 is identified as a novel protein required for full activation of Aurora B. Mechanistically, EHMT2 supports R‐loop accumulation at centromeres, promoting Aurora B activation through the ATR–CHK1 signalling pathway. Although R‐loops pose a genomic stability risk during the S‐phase, they are vital for accurate microtubule–kinetochore attachment and faithful chromosome segregation.^18^, ^51^ Further research is required to elucidate how EHMT2 sustains centromeric R‐loops.

EHMT2 is known to influence numerous signalling pathways, with its effects dependent on tumour progression, genetic background and interactions within the tumour microenvironment.^29^, ^52^ Elevated EHMT2 expression has been observed across various cancers and is associated with drug resistance and metastasis.^53^, ^54^, ^55^ EHMT2 inhibitors have shown efficacy in curbing the growth of certain cancer types, highlighting their potential as therapeutic agents. Although EHMT2's oncogenic role in PCa has recently been identified, its specific molecular mechanisms and regulatory dynamics in PCa remain poorly defined. This study reveals that EHMT2 interacts with the E3 ubiquitin ligase CUL3, which promotes EHMT2 protein degradation via ubiquitination. Notably, the EHMT2–CUL3 interaction weakens during mitosis, providing mechanistic insight into the CUL3/EHMT2 complex's role in spindle assembly and stability. It is proposed that cell cycle‐dependent regulation of the CUL3–EHMT2 interaction is essential for maintaining EHMT2 protein levels during mitosis, thereby enabling high‐CIN PCa cells to adapt to ongoing CIN. However, further investigation is needed to understand how these interactions are controlled by the cell cycle. Considering that EHMT2 and CUL3 are substrates of key mitotic kinases PLK1 and CDK1,^56^, ^57^ it is speculated that CDK‐dependent or PLK1‐induced phosphorylation of EHMT2 or CUL3 may regulate their interaction.

AR signalling is pivotal in PCa development, with Enz, a second‐generation AR inhibitor, widely used to treat patients with CRPC. However, resistance to Enz inevitably arises through various complex mechanisms, including AR‐related signalling pathways, glucocorticoid‐associated pathways, glycolysis and immune.^4^, ^38^, ^58^, ^59^, ^60^ This necessitates novel therapeutic strategies to combat Enz resistance. In CSPC, growth inhibition following AR suppression or a deprived of androgen predominantly results from a G1/S cell cycle arrest, regulated by AR‐dependent expression of cyclin D1 and p21.^61^ In contrast, CRPC displays selective upregulation of specific M‐phase cell cycle genes by AR, enabling tumour growth even under androgen‐deprived conditions.^62^ This phenomenon may partly clarify why maximum androgen blockade, integrating AR competitors and Luteinizing Hormone Releasing Hormone (LHRH)inhibitors, fails to improve survival in patients with CRPC^63^: such therapies target the androgen‐bound AR's function at the G1/S transition but do not prevent un‐liganded AR from promoting M‐phase progression in CRPC. Supporting this, clinical studies have revealed that docetaxel, which interrupts mitosis via preventing microtubule depolymerisation, offers a modest survival advantage in CRPC, suggesting the critical role of M‐phase processes in disease progression.^64^ This study identifies EHMT2 as a key player in Enz resistance through its activation of Aurora B, and that EHMT2 inhibition may counteract Enz resistance. Investigating the combined effects of EHMT2 inhibitors with AR signalling inhibitors could be valuable, potentially enhancing tumour‐cell eradication by targeting both G1/S and M‐phase transitions in CRPC.

## 4 CONCLUSIONS

In conclusion, this study identifies a regulatory pathway involving the CUL3–EHMT2–Aurora B axis as essential for accurate chromosome segregation, cell cycle progression, cell proliferation and ENZ resistance, highlighting EHMT2 as an intriguing candidate to conquer ENZ resistance and improve PCa prognosis.

## 5 MATERIALS AND METHODS

### 5.1 Cell lines and treatments

The PCa cells (22Rv1, DU145 and LNCaP, sourced from the Shanghai Institute of Life Sciences, Chinese Academy of Sciences) were grown in RPMI‐1640 or F12 medium (Dulbecco's modified Eagle's medium [DMEM]; Gibco). GFP‐H2B‐HeLa cells were grown in DMEM (Gibco). All media with 10% foetal bovine serum (FBS; Gibco) and 100 µg/mL streptomycin/penicillin. For cell cycle synchronisation, cells were arrested in the S‐phase or at the G1/S boundary via single or double treatment with 2.5 mM thymidine (Sigma), respectively, or in prometaphase using .66 µM nocodazole (Selleck). ENZ, monastrol, BIX‐01294, Dimethyl sulfoxide (DMSO) and MG132 were sourced from Selleck Chemicals or Sigma.

### 5.2 Cell transfection

For transient gene expression modulation, siRNA sequences targeting EHMT2, CHK1, Aurora B and CUL3 were sourced from (GenePharma). Transfections were conducted in accordance with the manufacturer's protocol using Lipofectamine RNAiMAX (Invitrogen).

For stable EHMT2 knockdown, lentiviral vectors carrying EHMT2‐targeting shRNA or control shRNA (sh‐NC) were procured, and stably infected cells were selected with puromycin. Western blotting was implemented to verify the expression of EHMT2 in the cell lines. The shRNAs and siRNAs utilised were as follows: si‐NC: UAGCGACUAAACACAUCAA; si‐EHMT2‐1: GCUCCAG GAAUUUAACAAGAU; si‐EHMT2‐2: CCAUGCUGUCAACUACCAUGG; si‐Aurora B: AGAGCTGCACATTTGACGA; si‐CHK1:GCAACAGUUAUUUCGGUAUAUU;si‐CUL3‐1: GAGAAGATGTACTAAATTC;si‐CUL3‐2: CGACAGAAAACATGAGATA; sh‐NC: CAACAAGATGAAGAGCACCAA; sh‐EHMT2‐1: CACACATTCCTGACCAGAGAT; sh‐EHMT2‐2: CGAGAGA GTTCATGGCTC TTT.

### 5.3 Immunofluorescence

Cells grown on coverslips were fixed in 4% paraformaldehyde in phosphate‐buffered saline (PBS) for 15 min, and subsequently permeabilised with .25% Triton X‐100 in PBS for 10 min. Primary antibodies were incubated with fixed cells for 2 h, followed by secondary antibodies for 1 h. Both antibodies were prepared in 1% bovine serum albumin (BSA) in PBS and were incubated at room temperature. DNA was counterstained with 4',6‐diamidino‐2‐phenylindole (DAPI) for 3–5 min. ImageJ was employed to analyse the data. The primary antibodies used for IF included human anti‐centromere antisera (Antibodies Inc., 15‐234‐0001), ATR (Bethyl Laboratories, A300‐138A), Aurora B (Abcam, ab287960), CHK1 (Santa Cruz Biotechnology, sc‐8408), INCENP (Novus Biologicals, NB100‐2286), p‐ATR‐T1989 (GeneTex, GTX128145), p‐Aurora B (Rockland, 600‐401‐677), p‐CHK1‐S317 (Cell Signaling Technology, 12302), p‐H3‐S28 (Cell Signaling Technology, 9713) and α‐Tubulin (Cell Signaling Technology, 3873).

### 5.4 Western blotting and immunoprecipitation (IP)

Cells were lysed using Radio Immunoprecipitation Assay (RIPA) buffer (Beyotime) combined with protein loading buffer (Solarbio). The extracted proteins were separated via sodium dodecyl‐sulphate polyacrylamide gel electrophoresis (SDS‐PAGE) gel and transferred to Polyvinylidene fluoride (PVDF) membranes, for immunoblotting. For immunoprecipitation, cells were lysed in Cell lysis buffer for Western and IP buffer (Beyotime) and lysates were incubated with antibodies for 12 h at 4°C, followed by the addition of Protein G Sepharose for an additional hour. Beads were then washed with IP buffer, boiled in loading buffer and analysed by immunoblotting. Primary antibodies for immunoblotting included EHMT2 (Cell Signaling Technology, 3306), α‐Tubulin (Cell Signaling Technology, 3873), phospho‐H3 S10 (Cell Signaling Technology, 53348), CUL3 (Cell Signaling Technology, 2759; Abcam, ab75851), β‐actin (Proteintech, 20536‐1‐AP), Ubiquitin (Cell Signaling Technology, 3936), phospho‐Aurora B (Rockland, 600‐401‐677) and Aurora B (Abcam, ab287960).

### 5.5 RNA extraction and RT‐PCR

RNA extraction was carried out with Trizol (Invitrogen), and quantitative RT‐PCR was conducted according to the manufacturer's procedure, with Glyceraldehyde‐3‐phosphate dehydrogenase (GAPDH) as a normalisation control. PCR primers were as follows: EHMT2, forward 5′‐TCCAATGACACATCTTCGCTG‐3′ and reverse 5′‐CTGATGCGGTCAATCTTGGG‐3′; GAPDH, forward 5′‐TGCACCACCAACTGCTTAGC‐3′ and reverse 5′‐GGCATGGACTGTGGTCATGAG‐3′.

### 5.6 Flow cytometry

For cell cycle analysis, cells were collected by trypsinisation, washed with 1 × PBS, and subsequently fixed in cold ethanol. Following 12 h incubation at 4°C, cells were pelleted and treated with RNase A, stained with PI. As per the directives, Annexin V‐PE Apoptosis Detection Kits (KeyGEN) were employed to assess apoptosis. Flow cytometry was conducted with a Cytomics FC500 (Beckman) with subsequent data analysis.

### 5.7 PCa tissue specimens and immunohistochemistry

A total of 20 primary and 18 metastatic PCa samples were collected from patients at The Third Affiliated Hospital of Soochow University from 2016 to 2018. Informed agreement was acquired from each participant, and those who had undergone systemic or local treatment were excluded from the research. The research received approval from The Third Affiliated Hospital of Soochow University. Immunohistochemistry was conducted to assess target protein expression following methods established in previous research. Immunohistochemistry images were collected utilising an Olympus BX63 microscopy, and immunostaining was scored by pathologists in a blinded manner. Primary antibodies for immunoblotting included EHMT2 (Cell Signaling Technology, 3306) and CLSTN2 (Proteintech, 21436‐1‐AP).

### 5.8 Xenograft animal mode

Male BALB/c nude mice sourced from The Third Affiliated Hospital of Soochow University and randomly assigned to experimental and control groups. For tumour growth analysis, 5 × 10^6^ DU145/22Rv1 cells with stable EHMT2 silencing were implanted into the flank of mouse. Tumour volumes were assessed weekly post‐injection and calculated according to the formula .5 × *a* ^2^ × *b*, where *a* and *b* represent the short and long tumour diameters, respectively. After 4 weeks, the mice were sacrificed, and tumours were excised, weighed and prepared for histological analysis and further studies.

### 5.9 Publicly available data

Datasets of GSE8511, GSE35988, GSE5803 and GSE114740 were obtained from the GEO database (http://www.ncbi.nlm.nih.gov/geo), EHMT2 expression in these datasets was analysed in the indicated groups. TCGA datasets (RNA‐seq) were obtained from (http://gdac.broadinstitute.org/).

### 5.10 Gene set enrichment analysis

To investigate the pathways of signalling related with EHMT2 expression in PCa, we divided samples from the TCGA‐PRAD datasets into two groups determined by the median expression level of EHMT2 and performed GSEA. Enrichment analysis was performed using gene sets downloaded from the Database (http://software.broadinstitute.org/gsea/index.jsp). |NES| > 1 and *p* < .05 were considered statistically significant.

### 5.11 RNA seq and bioinformatics analysis

To characterise the transcriptional program regulated by EHMT2, we performed RNA sequencing of Du145 cells, after 48 h of being transfected with Vector or EHMT2. After culturing, cells were collected, and total RNA extracted with the RNeasy kit. RNA quality was determined using Agilent TapeStation. Libraries were produced using the Illumina TruSeq RNA Kit and sequenced on the Illumina NovaSeq 6000 platform, resulting in 100‐bp single‐end reads. The raw sequencing reads were aligned with the *Homo sapiens* genome (GRCh37/hg19) from the NCBI. Gene expression levels were measured using featureCount in ‘count’ mode and normalised to transcripts per million with the R package ‘convertCounts’. DEGs were determined by the ‘DESeq2’. The analysis of functional enrichment of DEGs was conducted with the ‘clusterProfiler’.

### 5.12 DNA–RNA hybrid immunoprecipitation

Briefly, cells were transfected with RLAG‐NaseH1_D210N plasmids and cultured for 48 h before collection. SDS/proteinase K treatment at 37°C overnight separated DNA, which were then extracted with phenol–chloroform and precipitated with ethanol. Precipitated DNA were sonicated and then incubated with the FLAG antibody overnight at 4°C, then with BSA‐pretreated Protein G Beads (Invitrogen) for 2 h at 4°C. Antibody–DNA complex were wash and dissolved in Tris‐EDTA buffer (TE Buffer). Subsequently, quantitative PCR was conducted. The relevant primer sequences: Forward: ATGTTTGCATTCAAC TCACAGAG Reverse: CAACACAGTCCAAATATCCA GTTG. The fold change of the sample was computed after normalising the control sample to one. The background signal was eliminated by removing the ‘no antibody’ controls from the samples.

### 5.13 Statistical analysis

Data are presented as mean ± SD or SEM. Differences between two groups were analysed using the two‐tailed Student's *t*‐test for parametric data or the Mann–Whitney test for non‐parametric data. Prism was used for statistical analysis (*p < .05; **p < .01; ***p < .001).

## AUTHOR CONTRIBUTIONS

Dong Xue, Xiansheng Zhang and Xingliang Feng conceptualised the project. Yuyang Zhang, Mingqin Su, Yiming Chen, Li Cui, Wei Xia and Renfang Xu conducted or supervised the research. Yuyang Zhang and Xingliang Feng prepared the manuscript, with all other authors contributing to its editing.

## CONFLICT OF INTEREST STATEMENT

The authors declare no conflicts of interest.

## ETHICS STATEMENT

All animal experiments were conducted with the approval of The Third Affiliated Hospital of Soochow University (approval number CLO22H71). Patient samples were collected under ethical approval from The Third Affiliated Hospital of Soochow University Ethics Committee.

## Supporting information



Supporting Information

## Data Availability

Data are available from the corresponding author upon reasonable request.
