# Supplementary material for: EHMT2‐mediated R‐loop formation promotes the malignant progression of prostate cancer via activating Aurora B
Source: Clin Transl Med. 2025 Jan 6;15(1):e70164. doi: 10.1002/ctm2.70164 (PMC11705492; doi:10.1002/ctm2.70164)
Supplement: Supplementary file 1 — Supporting Information [file CTM2-15-e70164-s001.pdf]

Figure S1

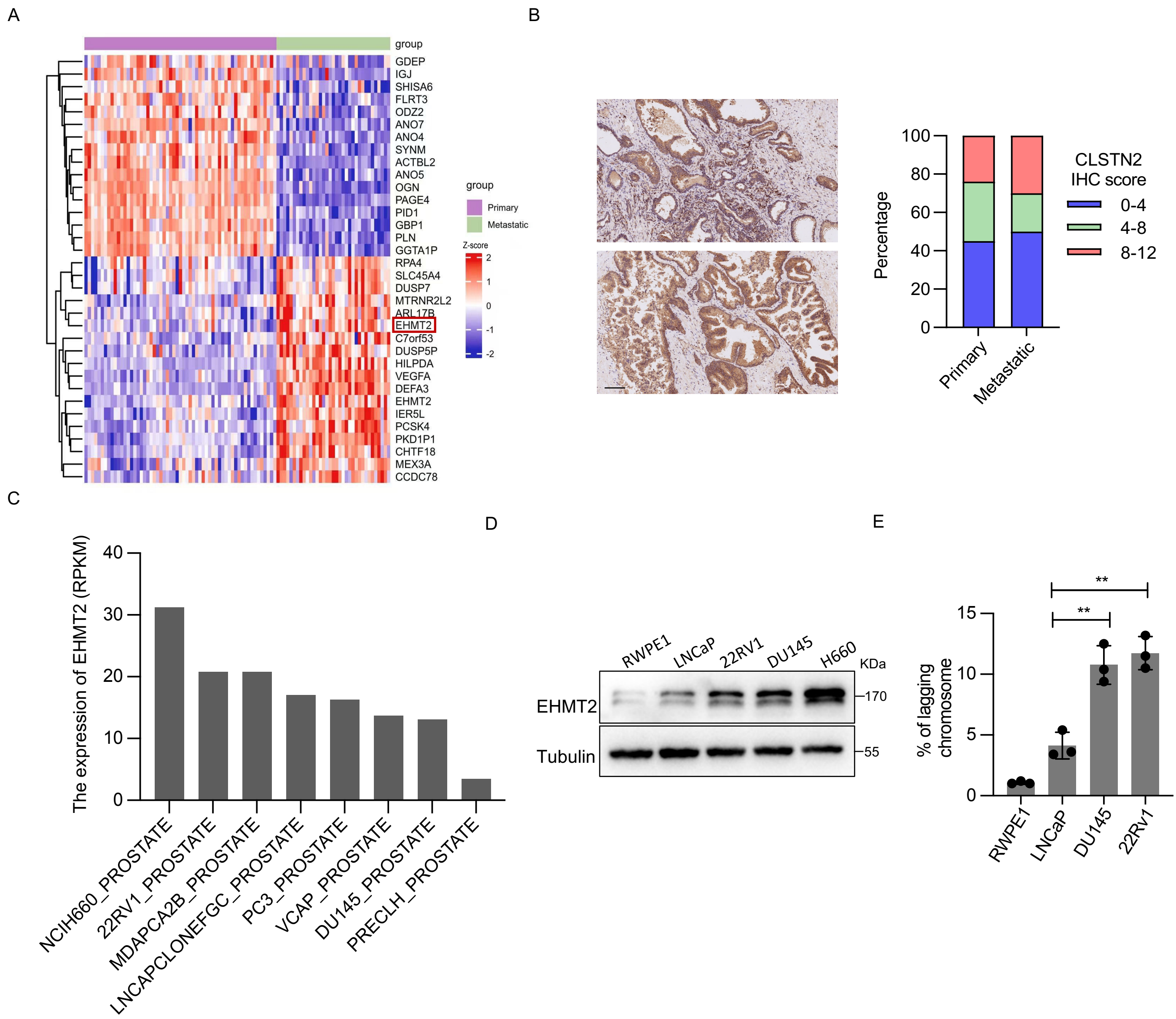

(A) Heatmaps display the differentially gene expression GSE5803. The red square highlights EHMT2.

(B) Representative CLSTN2 immunohistochemistry images and quantification of EHMT2 protein levels in metastatic (n = 20) and non-metastatic (n = 18) prostate cancer specimens. Scale bar, 100 μm.

(C) EHMT2 expression in different prostate cancer cell types was analyzed using the Cancer Cell Line Encyclopedia (CCLE) database.

(D) Analysis of EHMT2 protein expression in PCa cell lines and prostate epithelial cell line RWPE1.

(E) Percentage of lagging chromosomes in indicated prostate cell lines. **P<0.01.

Figure S2

A   B

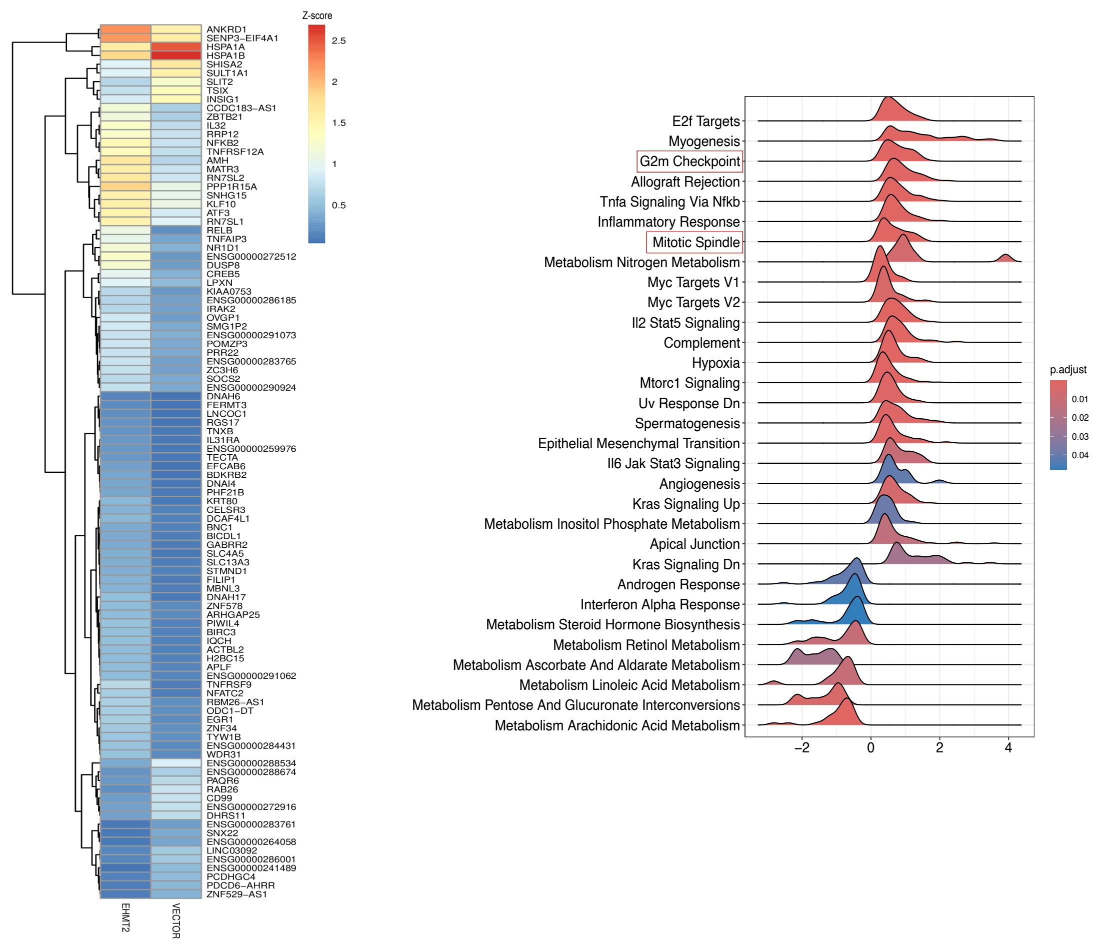

(A) Heat map of gene signature in DU145 cells transfected with Vector or EHMT2.
(B) GSEA revealed that EHMT2-related genes were enriched in multiple signaling pathways.

Figure S3

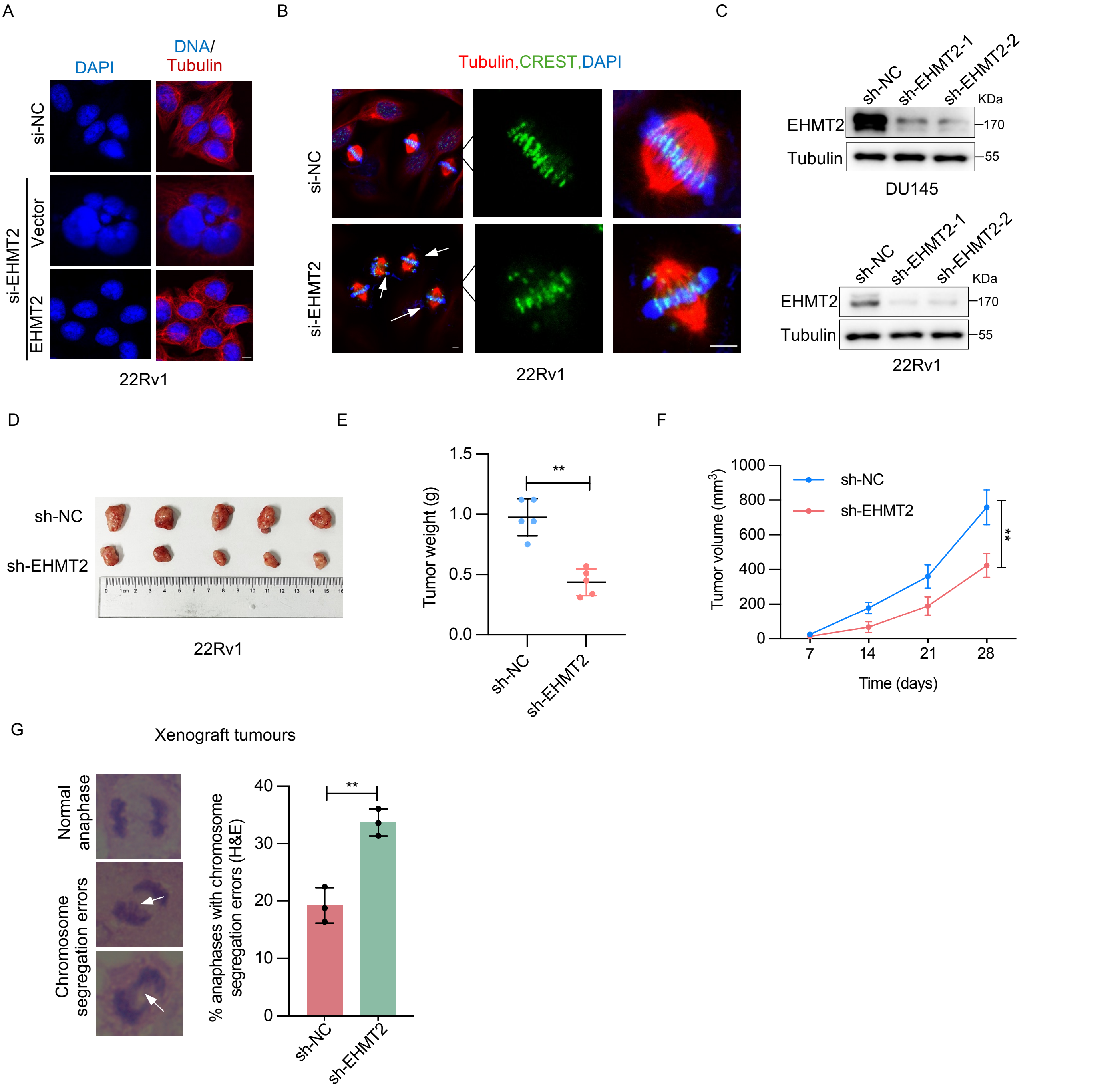

(A) Representative images of 22Rv1 cells transfected with either EHMT2 siRNA or control siRNA, subsequently were stained with DAPI and tubulin.

(B) Representative images of 22Rv1 cells transfected with either EHMT2 siRNA or control siRNA, subsequently were stained with DAPI, CREST and Tubulin.

(C) Western blot analysis of EHMT2 protein in DU145 and 22Rv1 cells with EHMT2 knockdown by shRNAs.

(D-F)Xenografts derived from 22Rv1-shEHMT2 and respective controls (n = 5). Tumor volume was recorded at indicated times to establish tumor weight (E) and growth curves (F).

(G) Representative images and quantification of anaphases with chromosome segregation errors in indicated xenograft tumors. Arrow points lagging chromosomes or chromatin bridges. **P<0.01.

Figure S4

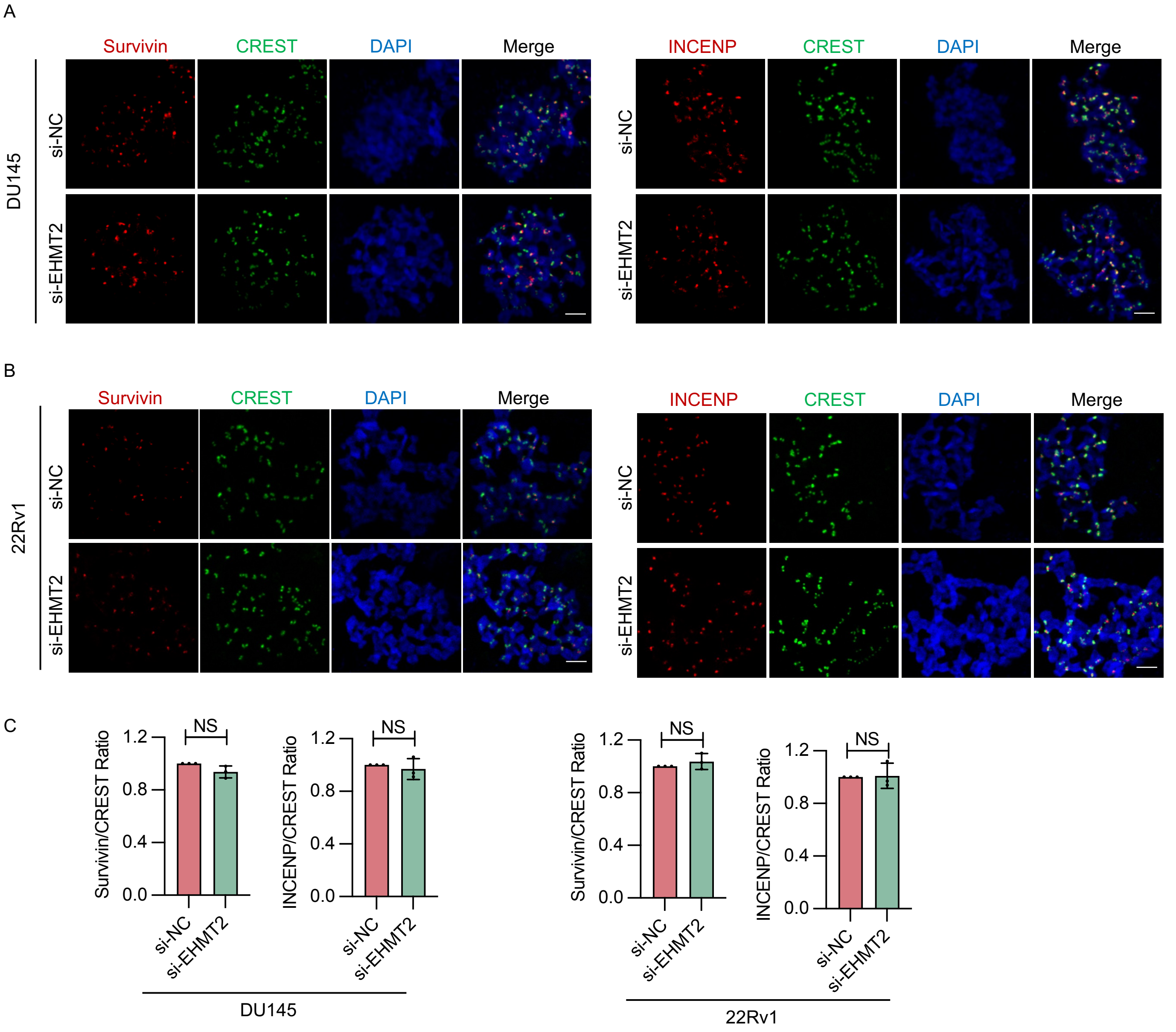

(A-C) Mitotic DU145 or 22Rv1 cells were stained with Survivin, INCENP and CREST. The quantification of signal intensity of Survivin or INCENP .Bars represent mean ± SEM from three independent experiments. NS: not significant.

Figure S5

A

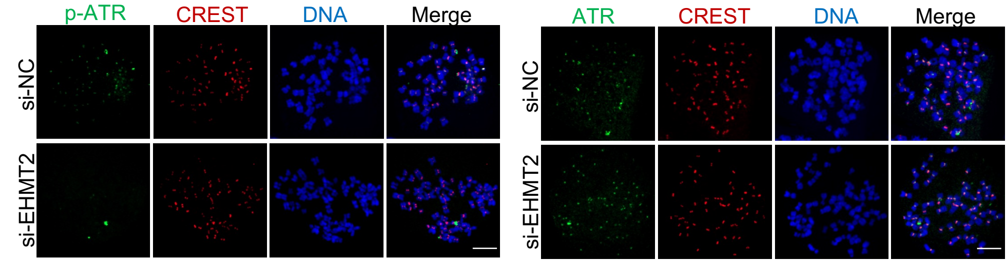

B

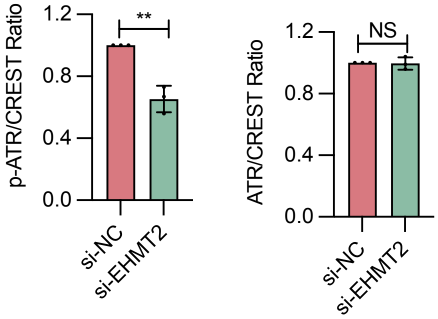

C

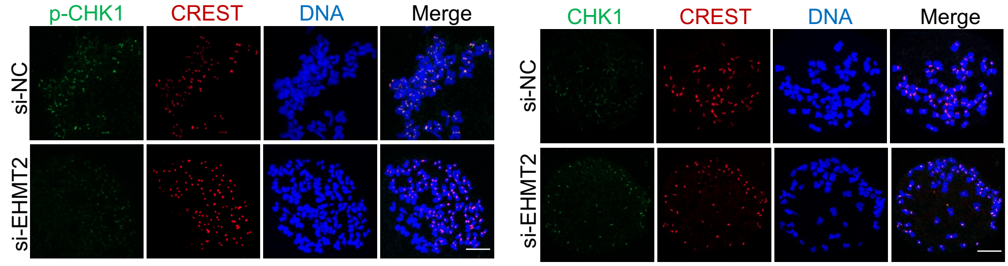

D

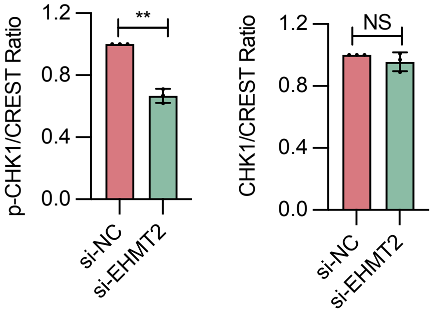

(A-D) Mitotic 22Rv1 cells were stained with p-ATR, ATR, CHK1, p-CHK1 and CREST. Bars represent mean ± SEM from three independent experiments. NS: not significant; *P < 0.05, **P<0.01.

Figure S6

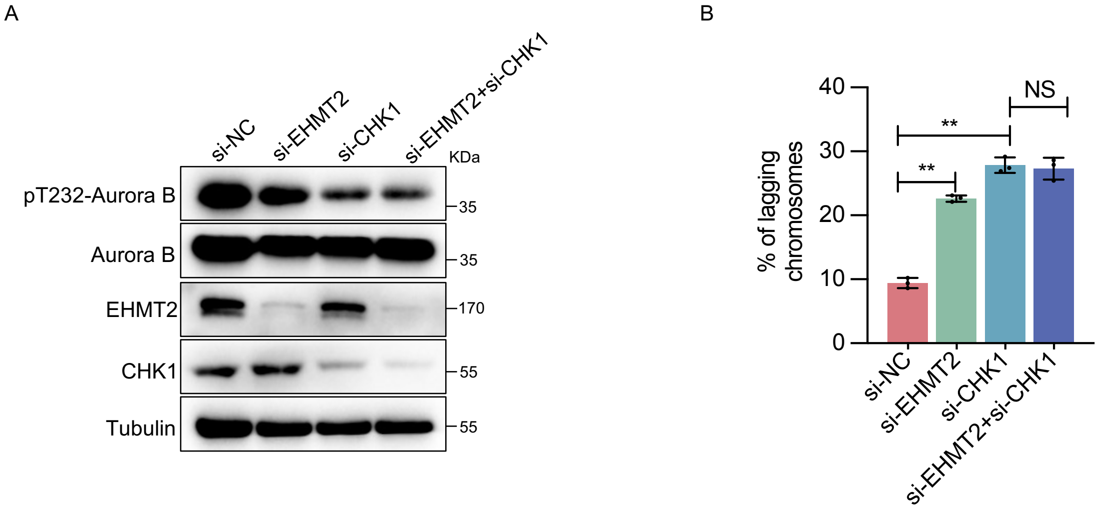

(A) Immunoblotting of mitotic DU145 cell lysates obtained from cells transfected indicated siRNA.
(B) Quantification of lagging chromosomes phenotypes in cells transfected with indicated siRNAs. Error bars represent Mean±S.E.M from three independent experiments. NS: not significant; **P<0.01.

Figure S7

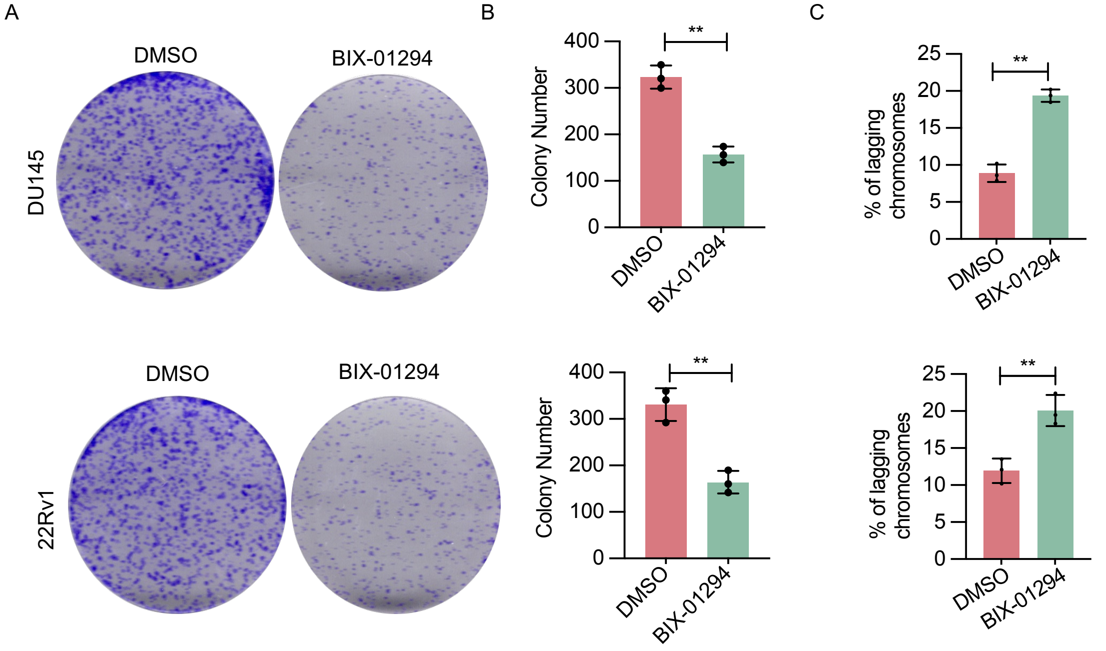

(A-B) DU145 and 22Rv1 cells were treated with DMSO or BIX-01294. Colonogenic assay was performed, and colony numbers were quantified.
(C) Quantification of lagging chromosomes phenotypes in treated with DMSO or BIX-01294. Error bars represent Mean±S.E.M from three independent experiments. **P<0.01.

Figure S8

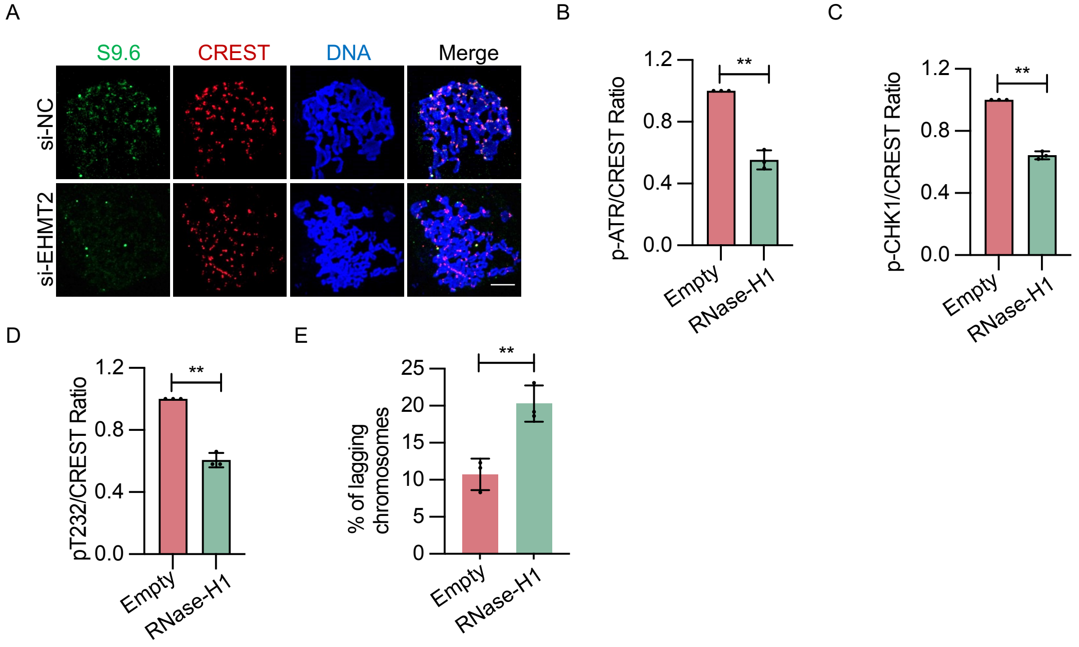

(A) Representative images of centromeric R-Loop in mitotic DU145 cells. Cells were treated with nocodazole for 6 hours to promote chromosome spreading.
(B-D) Fluorescence intensity of centromeric p-ATR,p-CHK1 and p-Aurora B in cells transfected empty or RNaseH1-WT vector.
(E) Quantification of lagging chromosomes phenotypes in transfected with Empty or RNase-H1. Error bars represent Mean±S.E.M from three independent experiments. NS: not significant; *P < 0.05, **P<0.01.

Figure S9

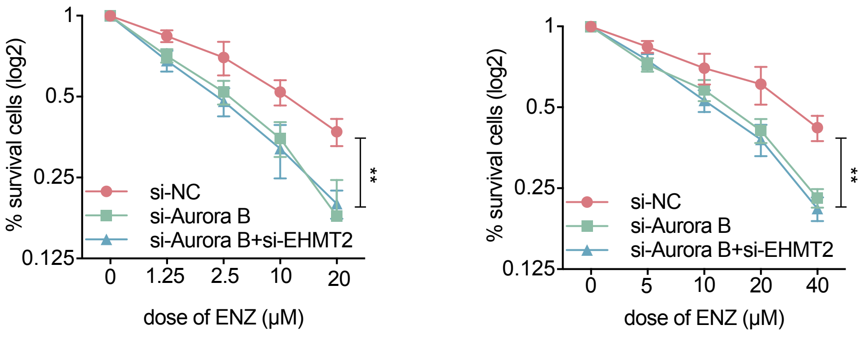

Control LNCaP/22Rv1 cells and engineered LNCaP/22Rv1 cells (transfected with indicated siRNAs) were treated with increasing doses of enzalutamide, and the cell survival rate was assessed by CCK-8 analysis. NS: not significant; *P < 0.05, **P<0.01.
